# Efficacy of radical reactions of isocyanides with heteroatom radicals in organic synthesis

**DOI:** 10.3762/bjoc.20.182

**Published:** 2024-08-26

**Authors:** Akiya Ogawa, Yuki Yamamoto

**Affiliations:** 1 Organization for Research Promotion, Osaka Metropolitan University, 1-1 Gakuen-cho, Nakaku, Sakai, Osaka 599-8531, Japan; 2 Graduate Faculty of Interdisciplinary Research, University of Yamanashi, 4-4-37 Takeda, Kofu 400-8510, Japan

**Keywords:** aza-Bergman cyclization, heteroatom-mixed system, imidoyl radical, isocyanide, radical addition, radical cyclization

## Abstract

Isocyanide is a promising synthetic reagent not only as a one-carbon homologation reagent but also as a nitrogen source for nitrogen-containing molecules. Because of their isoelectronic structure with carbon monoxide, isocyanides also react with nucleophiles, electrophiles, carbon radicals, and transition metal reagents, and are widely used in organic synthesis. On the other hand, the use of isocyanides in reactions with heteroatom radicals is limited. However, the reaction of isocyanides with heteroatom radicals is a promising synthetic tool for the construction of nitrogen-containing organic molecules modified with a variety of heteroatoms. In this Perspective, we review the addition and cyclization reactions of heteroatom radicals with isocyanides and discuss the synthetic prospects of the reaction of isocyanides with heteroatom radicals.

## Introduction

**Figure 1:**
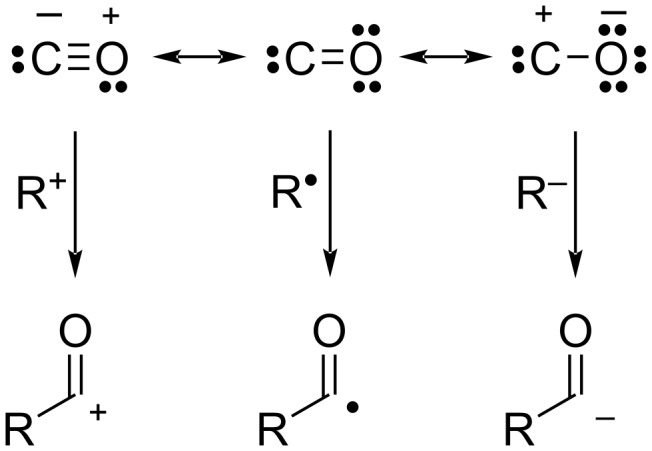
Resonance structures and reactivity of carbon monoxide.

Carbon monoxide is a very important C1 resource in both synthetic and industrial chemistry and is not only capable of reacting with a variety of active species such as carbon cations, carbon anions, and carbon radicals (Figure 1), but is also widely used in transition-metal-catalyzed carbonylation reactions [1–2]. However, carbon monoxide is a flammable gas with a wide explosive range, although colorless and odorless, and requires special care in handling due to its high toxicity. In addition, when carbon monoxide is used in a reaction, pressurization in an autoclave or other pressurization device is required to increase the CO concentration.

**Figure 2:**
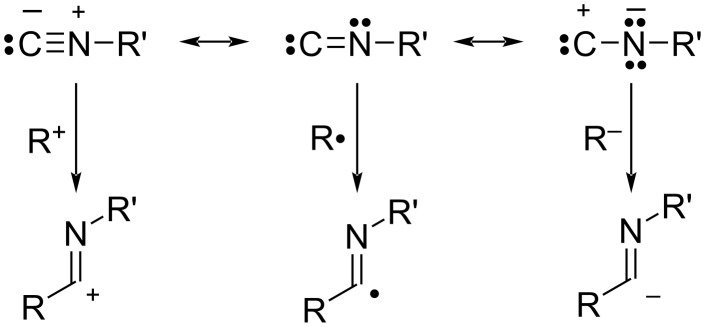
Resonance structures and reactivity of isocyanides.

Isocyanides, on the other hand, have an isoelectronic structure with carbon monoxide and are expected to be not only a promising C1 resource but also an important synthetic reagent for nitrogen-containing compounds [3–7]. Furthermore, by adjusting the substituents on the nitrogen, reactivity can be controlled and solubility in various solvents can be tuned (Figure 2).

However, the use of isocyanide as a C1 resource is somewhat limited compared to that of carbon monoxide [8] because isocyanide is susceptible to multiple imidoylation [9–11], whereas carbon monoxide is less susceptible to multiple carbonylation. Therefore, precise control of the reaction is required for selective formation of the monoimidoylation product.

Regarding the radical reaction of isocyanides, the reaction of carbon radicals with isocyanides generates imidoyl radicals as key active species [12], and addition and cyclization reactions using these radical species are useful in synthetic organic chemistry, especially multicomponent synthesis. If various functional groups can be appropriately attached to imidoyl units generated in situ by radical addition to isocyanides, innovative molecules with a variety of functions can be obtained. In other words, if functional groups can be prepared simultaneously with the formation of an imidoyl group, it would be an extremely useful method for the synthesis of nitrogen-containing functional molecules. To achieve this goal, it is expected to be effective to develop a new method to react heteroatom radicals with isocyanides to generate imidoyl radicals to which various heteroatom groups are attached and to use them as synthetic reagents. However, systematic studies of addition reactions of heteroatom radicals to isocyanides are still limited. In this perspective paper, we systematically review the addition reactions of heteroatom radicals to isocyanides and discuss prospects.

## Discussion

### 1,1-Addition of heteroatom radicals to isocyanides

#### Generation of heteroatom radicals

When reacting a heteroatom radical with an isocyanide, the first thing to consider is the method of generating the heteroatom radicals [13–15]. As mentioned above, isocyanides are readily polymerizable molecules, so to suppress the polymerization of isocyanides, it is necessary to understand the conditions for the generation of heteroatom radicals. In addition, from the perspective of recent green chemistry, the development of environmentally friendly synthetic methods is strongly demanded. In other words, a new synthetic method should have excellent atom economy, produce no waste, be aware of resource recycling, and promote the use of natural energy [16].

**Scheme 1:**
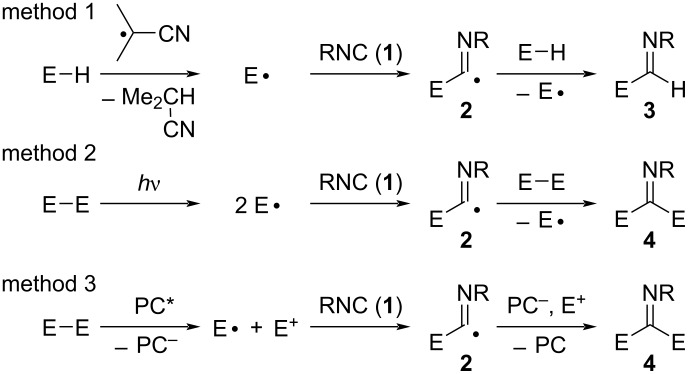
Possible three pathways of the E• formation for imidoylation.

The following three methods are generally used to generate heteroatom radicals (E•) (Scheme 1). In method 1, E• is generated by hydrogen abstraction from E–H by cyanoisopropyl radicals generated by thermal decomposition of 2,2'-azobis(isobutyronitrile) (AIBN). Then, E• adds to isocyanide **1** to form imidoyl radical **2**, which abstracts hydrogen from E–H. The addition reaction proceeds by a radical chain mechanism, producing the 1,1-addition product **3** with regeneration of E•. In method 2, E• is generated by homolysis of a heteroatom–heteroatom bonded compound (E–E) upon heating or photoirradiation. Similarly, E• adds to **1** to form **2**, which undergoes atom (or group)-transfer from E–E to give the 1,1-addition product **4** with regeneration of E• [17–18]. In method 3, the photoinduced redox reaction of a heteroatom compound takes place using metal complex or functional dye as a photocatalyst (PC) [19–20]. Recently, some important reviews summarize and discuss the use of method 3 in synthetic organic chemistry [21–22]; in contrast, there is little detailed and coherent literature on the overall research trends regarding the latest research on molecular transformations by the reactions of heteroatom radicals with isocyanides using methods 1 and 2. Thus, in this perspective, we mainly focused on the use of method 1 and method 2 for the generation of heteroatom radicals and the reactivity of them with isocyanides.

Among these three methods, methods 1 and 3 require the addition of a radical initiator and a photocatalyst, respectively. In contrast, method 2 does not require any additive, although, if the heating or photoirradiation is performed by electricity, the combustion of fossil fuels may cause environmental pollution. However, when using sunlight, which is an inexhaustible natural energy, it is expected to be the most environmentally friendly method.

The homolysis of E–E upon visible light irradiation is induced by exciting one electron of the isolated electron pair on E to the anti-bonding orbital of the E–E-bond (σ* orbital). Such an n–σ* transition usually indicates the maximum absorption in the near-UV region, and the absorption cutoff reaches the visible region, especially in the case of highly periodic E–E. Therefore, interelement compounds E–E with groups 15–17 heteroatoms have isolated electron pairs, and E• can be generated by photoirradiation. On the other hand, the photoinduced homolysis of groups 13 and 14 interelement compounds with B–B, Si–Si, Sn–Sn bonds, etc. is generally impossible, because such E–E compounds have no isolated electronic pair. Therefore, the use of a photocatalyst (method 3) or the combination with groups 15–17 interelement compounds would be considered effective for the generation of groups 13 and 14 heteroatom radicals.

#### Radical addition of group 17 compounds to isocyanides

If isocyanides (RNC) can undergo a radical addition of hydrogen halides (HX) and molecular halogens (X_2_), 1,1-addition products RN=CH-X, **3** (E = X: F, Cl, Br, I) and RN=CX_2_, **4** (E = X), respectively, could be formed. In practice, however, very few examples of such radical addition to isocyanides are known, and the 1,1-addition products **3** and **4** have usually been synthesized by ionic reactions. 1,1-Addition of molecular bromine to phenyl isocyanide was reported by E. Kühle et al. to afford the corresponding 1,1-adduct (PhN=CBr_2_) [23]. Since dichloro compounds (RN=CCl_2_) [24] are the imino derivatives of highly toxic phosgene (O=CCl_2_), reactions using them as key intermediates are not safe synthetic methods. For these reasons, it is no exaggeration to say that radical reactions of group 17 interelement compounds with isocyanides have hardly been developed.

Upon exposure to near-UV light, perfluoroalkyl iodides (R_F_I) undergo homolysis to form perfluoroalkyl radicals (R_F_•) and iodine radical (I•). The perfluoroalkyl radical, as a carbon radical, rather than iodine radical can add to isocyanides to form imidoyl radicals. Then, the iodine atom of R_F_I can trap the imidoyl radicals to give the corresponding 1,1-adducts (R–N=C(I)–R_F_) in good yields [25–26].

#### Radical addition of group 16 compounds to isocyanides

**Scheme 2:**
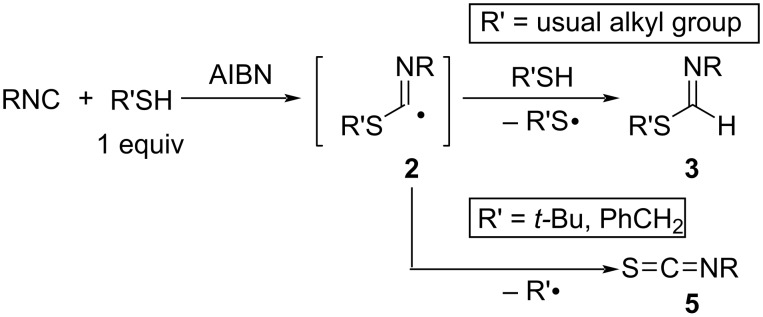
Radical addition of thiols to isocyanides.

In a pioneering study, Ito and Saegusa et al. reported the radical addition of thiols to isocyanides (Scheme 2) [27]. Thermal decomposition of AIBN as a radical initiator generates the 2-cyano-2-propyl radical ((NC)(CH_3_)_2_C•), which abstracts hydrogen from the thiol (R’SH) to form the thiyl radical (R’S•). The formed R’S• adds to isocyanide (RNC) to generate imidoyl radical intermediate **2** (E = R’S), which abstracts hydrogen from thiol to give the corresponding thioformimidate **3** (E = R’S) with regeneration of R’S•. Thus, the hydrothiolation of isocyanides with thiols proceeds by the radical chain mechanism. In the case of tertiary alkanethiols and arylmethanethiols, the corresponding imidoyl radicals **2** decompose to give tertiary alkyl and benzylic radicals, respectively, to form isothiocyanates **5**.

On the other hand, we have investigated the radical addition of diphenyl disulfide to isocyanides under photoirradiation. The photoinduced radical addition of the disulfide to aliphatic isocyanides hardly proceeds, whereas the radical addition to aromatic isocyanides proceeds under high concentration conditions using excess amounts of (PhS)_2_, selectively yielding 1,1-addition product **4** (R = 2,6-xylyl, E = PhS) [28]. In the case of aromatic isocyanides, the 1,1-addition reaction is probably more likely to proceed because the C–N double bond of the 1,1-addition product **4** (R = Ar, E = PhS) is conjugated to the aromatic ring, which stabilizes it compared to the corresponding adduct with aliphatic isocyanides. Because of this conjugation, the aromatic ring at the N of the 1,1-addition product **4** (R = Ar, E = PhS) geometrically isomerizes faster than the NMR timescale, so that the two thio groups of **4** are observed to be equivalent in NMR spectroscopy.

In the case of diphenyl diselenide as a representative organic diselenide, the addition of PhSe• to alkenes proceeds 10 to 50 times slower than PhS• [29]. For this reason, the addition of (PhSe)_2_ to isocyanides, whether aliphatic or aromatic, rarely proceeds. The exception is the addition to *p*-nitrophenyl isocyanides, which does proceed, but this is because the electron-withdrawing group improves the stability of the product **4** (R = *p*-O_2_N-C_6_H_4_, E = PhSe) and also because phase separation is caused by the product precipitation from the reaction solution. On the other hand, the addition reaction of (PhTe)_2_ to isocyanides does not proceed at all [30]. This is because the addition product **4** (E = PhTe) is unstable under photoirradiation conditions.

**Scheme 3:**
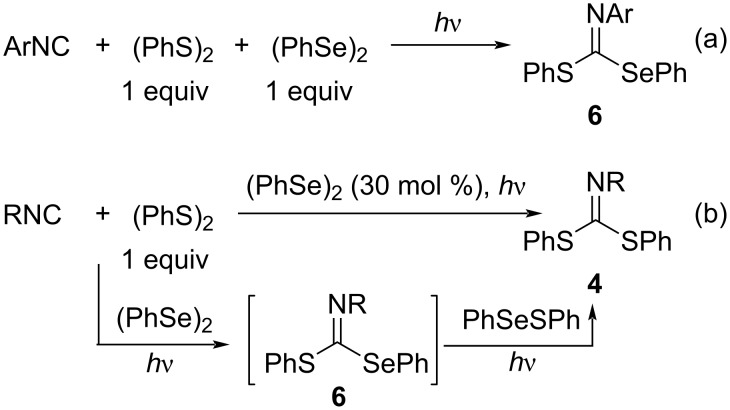
Selective thioselenation and catalytic dithiolation of isocyanides.

The photoinduced 1,1-dithiolation reaction of isocyanides required an excess of (PhS)_2_ due to the low carbon radical capturing ability of (PhS)_2_ (*k*_S_ = 7.6 × 10^4^ M^−1^s^−1^). In contrast, the carbon radical capturing ability of (PhSe)_2_ (*k*_Se_ = 1.2 × 10^7^ M^−1^s^−1^) is known to be ca. 160 times higher than that of (PhS)_2_ [31]. Therefore, we investigated the radical addition to isocyanides using a disulfide–diselenide binary system under photoirradiation and found that the thioselenation of aromatic isocyanides proceeded efficiently to afford the corresponding thioselenation products **6** (Scheme 3a).

More interestingly, it was found that the thioselenation of aliphatic isocyanides also proceeds in the initial stage of the reaction, but the formed thioselenation products **6** were gradually converted to the dithiolation products **4** under photoirradiation conditions (Scheme 3b). Thus, it was shown that the photoirradiated dithiolation of aliphatic isocyanides with (PhS)_2_ proceeded as a catalytic reaction of (PhSe)_2_ (30 mol %). In the dithiolation products **4** from aliphatic isocyanides, two PhS-groups were observed non-equivalently, suggesting the lack of geometrical isomerization of the C–N double bond of the dithiolation products [32].

**Scheme 4:**
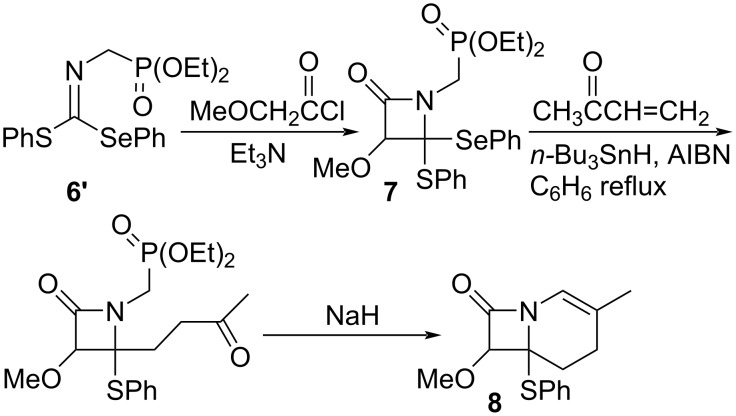
Synthesis of carbacephem framework.

The thioselenation product **6’**, an imine derivative, can be converted to a β-lactam derivative by [2 + 2] cycloaddition with ketene generated in situ. For example, thioselenation of RNC (R = (EtO)_2_P(O)–CH_2_) gave imine **6’** (96%), which underwent [2 + 2] cycloaddition with methoxyketene to afford β-lactam derivative **7** (79%) (Scheme 4). Selective replacement of the PhSe group of **7** with a 3-butanonyl group (34%) and the subsequent intramolecular Horner–Emmons reaction successfully led to carbacephem skeleton **8** (96%).

Since it is known that the carbon radical capturing ability of (PhTe)_2_ is even four times higher than that of (PhSe)_2_ [31], we investigated the radical addition to isocyanides using a disulfide–ditelluride binary system under photoirradiation. The visible-light-irradiated thiotelluration reaction did not proceed at all for normal isocyanides, but for aromatic isocyanides with electron-withdrawing groups (EWG) such as *p*-NO_2_, *p*-CF_3_, *p*-CN, *p*-Cl, and *m*-MeO, the desired thiotelluration reaction proceeded under visible light irradiation to successfully afford the corresponding thiotelluration products **4** (R = EWG-C_6_H_4_, E = PhS and PhTe) in moderate to high yields [30].

**Scheme 5:**
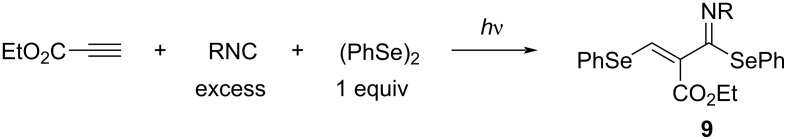
Sequential addition of (PhSe)_2_ to ethyl propiolate and isocyanide.

Very few examples of intermolecular cascade reactions with imidoyl radicals as key intermediates have been reported. The intermolecular cascade reaction of diselenide, electron-deficient acetylene, and isocyanide under photoirradiation yields the sequential addition products **9** in moderate to excellent yields (Scheme 5) [33]. The product can be used as a precursor for the carbapenem scaffold, one of the basic scaffolds of antibiotics.

**Scheme 6:**
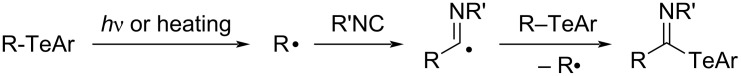
Isocyanide insertion reaction into carbon-tellurium bonds.

Thermal or photoirradiated decomposition of organotellurium compounds generates carbon radicals that can add to isocyanides to form the imidoyl radicals. In this reaction, telluro radicals (ArTe•) also forms in situ, but the relative reactivity of them toward isocyanides might be very low. In addition, dimerization of ArTe• to (ArTe)_2_ is very fast, and therefore, the ArTe-substituted imidoyl radical (ArTe-C•(=NR’)) could not be observed. However, the tellurium group of RTeAr can successfully trap the imidoyl radicals to yield the corresponding isocyanide-inserted organotellurium compounds (Scheme 6) [34–36].

#### Radical addition of group 15 compounds to isocyanides

Tetrakis(trimethylsilyl)hydrazine ((Me_3_Si)_2_N–N(SiMe_3_)_2_, **10**) undergoes ultraviolet light-induced homolysis of the N–N single bond of **10** to form bis(trimethylsilyl)aminyl radical ((Me_3_Si)_2_N•, **11**). The aminyl radical **11** adds to *tert*-butyl isocyanide to form the corresponding imidoyl radical **2** (R = *t*-Bu, E = (Me_3_Si)_2_N), as confirmed by ESR measurement [37].

**Scheme 7:**
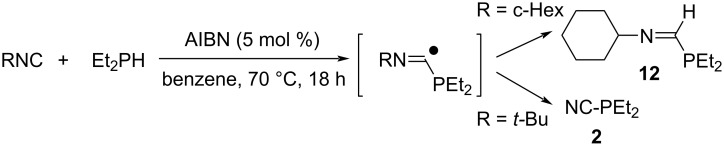
Radical addition to isocyanides with disubstituted phosphines.

Similar to the thiol addition to isocyanides, disubstituted phosphines (R’_2_PH) induce radical addition to isocyanides in the presence of AIBN as a radical initiator yielding the corresponding iminoformyl phosphines, R’_2_P–CH=NR, (**12**, R = c-C_6_H_11_ or *n*-C_6_H_11_, E = Et_2_P) in good yields. In the case of *tert*-butyl and benzyl isocyanides, the substituents on the nitrogen of imidoyl radical **2** (R = *t*-Bu or PhCH_2_, E = Et_2_P or Ph_2_P) were eliminated to give cyanophosphines (R’_2_P–CN, Scheme 7) [38].

**Scheme 8:**
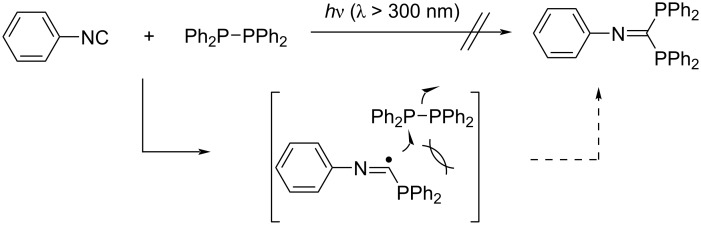
Radical addition to phenyl isocyanides with diphosphines.

On the other hand, we attempted a photoinduced addition of phosphorus–phosphorus interelement compounds such as Ph_2_P–PPh_2_ and Ph_2_P(S) –PPh_2_ to phenyl isocyanide, but the addition did not proceed at all. This is most likely due to the bulkiness of the Ph_2_P and Ph_2_P(S) groups (Scheme 8) [39].

In sharp contrast, the addition of a germyl phosphine (Et_2_P–GeEt_3_) to phenyl isocyanide was reported to give the corresponding 1,1-adduct (Et_2_P–C(=NPh)–GeEt_3_, **13**) in 46% yield [40]. Similarly, Me_2_N-SnMe_3_ was known to add to *p*-tolyl isocyanide to give Me_2_N–C(=N–C_6_H_4_–*p*-Me)–SnMe_3_ (**14**) in good yield [41]. However, the authors did not specify whether the addition reactions proceeded by a radical or ionic mechanism.

#### Radical addition of group 14 compounds to isocyanides

**Scheme 9:**
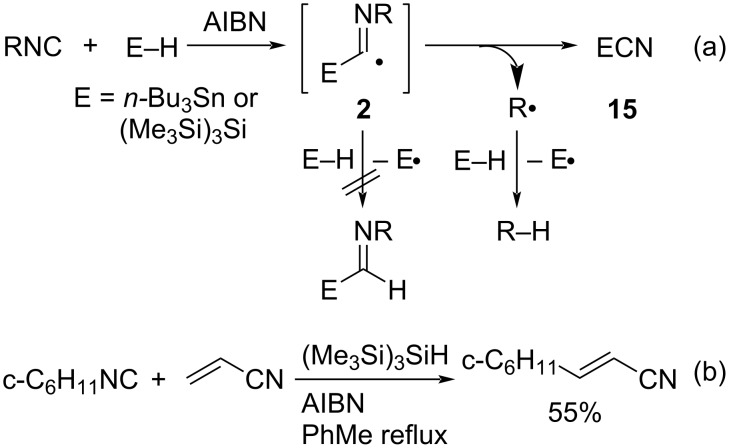
Radical reaction of tin hydride and hydrosilane toward isocyanide.

Group 14 compounds with an E–H or E–E bond (E = Si, Ge, Sn) have no lone-pair electrons and therefore cannot generate group 14 heteroatom radicals by homolysis via the n–σ* transition. To generate group 14 heteroatom radicals, the hydrogen abstraction reaction from tin hydride or hydrosilane by radical initiators such as AIBN has effectively been used. When tin and silyl radicals generated in this way are reacted with isocyanides, they are more susceptible to steric hindrance than group 16 or 15 heteroatom radicals due to the greater number of substituents on the heteroatom. For this reason, the 1,1-addition is less likely to proceed as with group 16 or 15 heteroatom radicals. In the case of stannyl and silyl radicals, the alkyl group of the isocyanide is eliminated as an alkyl radical from the imidoyl radical intermediate **2** [42]. The formed alkyl radicals abstract hydrogen from the tin hydride or hydrosilane, and the reduction reaction proceeds with the concomitant formation of stannyl or silyl cyanide **15** as byproducts (Scheme 9a) [38,43].

In the presence of acrylonitrile, the formed alkyl radical can add to acrylonitrile, affording the addition product (c-C_6_H_11_CH_2_CH_2_CN) after hydrogen abstraction from tris(trimethylsilyl)silane (TTMSS, (Me_3_Si)_3_SiH) (Scheme 9b) [44]. Triethylsilane (Et_3_SiH), one of the most popular hydrosilanes, has a strong Si–H bond (90 kcal/mol), and therefore the radical-chain reaction using Et_3_SiH is often difficult to perform. In contrast, tris(trimethylsilyl)silane, (Me_3_Si)_3_SiH, has a bond dissociation energy similar to that of *n*-Bu_3_SnH (74 kcal/mol) and can be used as an efficient reducing agent/mediator.

#### Radical addition of group 13 compounds to isocyanides

**Scheme 10:**
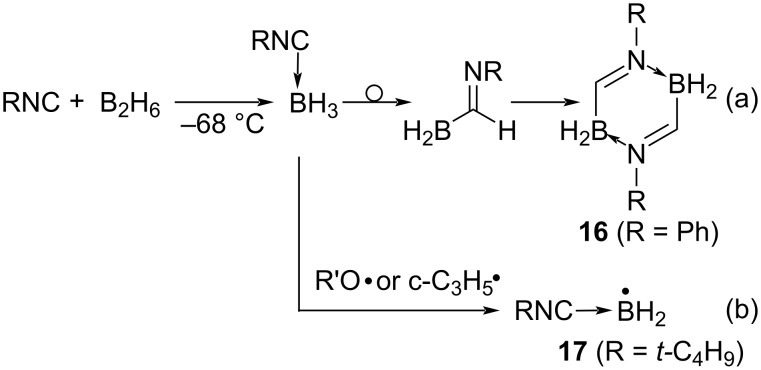
Isocyanide insertion into boron compounds.

Boron, a group 13 typical element, also lacks a non-covalent electron pair, making it impossible to generate boron radicals by homolysis via the n–σ* transition. In addition, since boron has an empty orbital, it forms ate complexes when Lewis base compounds coexist. As the result, the boryl groups of the ate complexes are bulky and often cause steric hindrance. However, several examples of isocyanide insertion reactions into B–H and B–B bonds are known. For example, isocyanides coordinate to diborane (B_2_H_6_) or trialkylboranes (BR'_3_) to form Lewis acid–base complexes (RNC→BH_3_ or RNC→BR'_3_), but these complexes are thermally labile, and hydrogen or alkyl groups on boron are 1,2-shifted to the isocyanide carbon, yielding the compounds (H_2_B-C(=NR)-H or R'_2_B-C(=NR)-R') with the isocyanide inserted between the B–H or B–alkyl bond [45–46]. The insertion products easily underwent dimerization to afford 2,5-diboradihydropyrazine derivatives **16** (Scheme 10a). Since this 1,2-shift reaction proceeds under mild conditions and in the absence of a radical initiator, it is thought to proceed by an ionic rather than a radical mechanism.

Alkoxy and cyclopropyl radicals, which are more reactive than the usual alkyl radicals, are capable of abstracting hydrogen from Lewis acid–base complexes (*t*-BuNC→BH_3_) to generate the corresponding isocyanide–boryl radicals **17** (*t*-BuNC→BH_2_•), which can be observed by ESR (Scheme 10b) [47]. However, the synthetic application of this boryl radical has not been investigated.

**Scheme 11:**
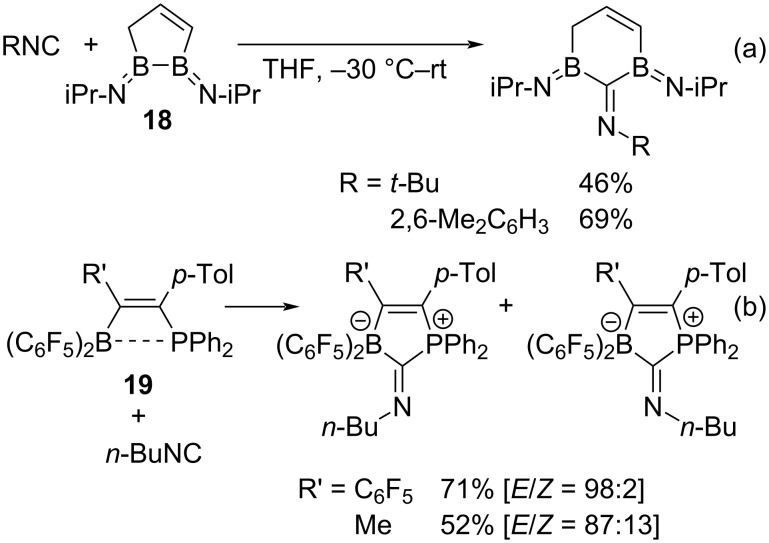
Isocyanide insertion into cyclic compounds containing boron units.

Among the cyclic diboron compounds, a series of five-membered cyclic diboron compounds **18** undergo an insertion reaction of isocyanides into the boron–boron single bond of **18** under mild conditions without the addition of any additives (Scheme 11a) [48]. The reaction is thought to proceed by an ionic mechanism.

Recently, several insertion-type reactions of isocyanides into diboron compounds have been reported to proceed by an ionic mechanism [49–50]. As an interesting example, a frustrated Lewis ion pair **19** consisting of a boryl and a phosphinyl group undergoes an isocyanide insertion reaction (Scheme 11b) [51–52]. As described above, the isocyanide insertion reaction into B–H or B–B bonds has been reported, but the reactions by a radical mechanism are largely unknown.

**Scheme 12:**
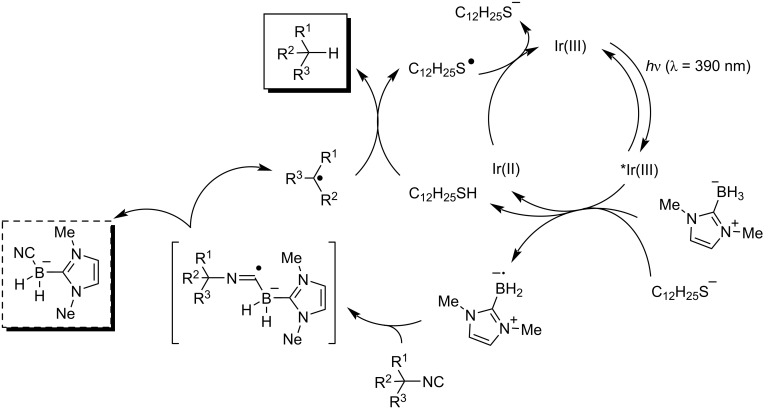
Photoinduced hydrodefunctionalization of isocyanides.

Very recently, Turlik and Schuppe reported a novel generation of nucleophilic boryl radicals using hydrogen atom transfer (HAT) and photoredox catalysis. Furthermore, its reaction with isocyanides forms boron-substituted imidoyl radical intermediates and rapid β-scission then causes elimination of the substituents on the nitrogen (Scheme 12) [53].

### Radical cyclization via formation of imidoyl radical species

In the former chapter, we discussed 1,1-addition reactions of typical element compounds to isocyanides using imidoyl radicals as key intermediates. However, as the number of substituents on typical elements increases, intermolecular 1,1-addition reactions become more difficult due to the increase in steric hindrance. Therefore, it is expected that intramolecular cyclization of imidoyl radicals will be possible by introducing an unsaturated group at an appropriate position in the isocyanide molecule, since intramolecular reactions are generally 10^3^ times faster than intermolecular reactions. This chapter discusses the intramolecular radical cyclization reactions of isocyanides with alkenyl, alkynyl, aryl, and isocyano groups as unsaturated groups.

#### Intramolecular cyclization of *ortho*-alkynylaryl- or *ortho*-alkenylaryl isocyanides

**Scheme 13:**
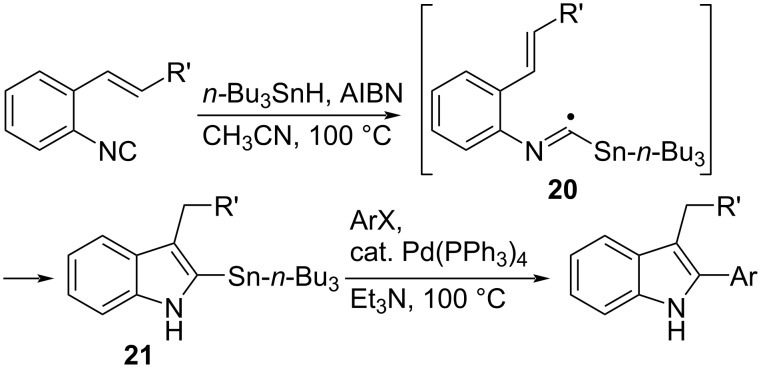
Tin hydride-mediated indole synthesis and cross-coupling.

Fukuyama et al. reported that the reaction of an aryl isocyanide with an alkenyl group at the *ortho*-position with tin hydride in the presence of AIBN generates a stannylated imidoyl radical **20**. The subsequent 5-*exo* cyclization, hydrogen abstraction from *n*-Bu_3_SnH, and aromatization successfully afforded the stannylated indole derivative **21** (Scheme 13) [8,54–56]. The stannyl group of **21** could be transferred to aryl or vinyl group by cross-coupling reaction.

**Scheme 14:**
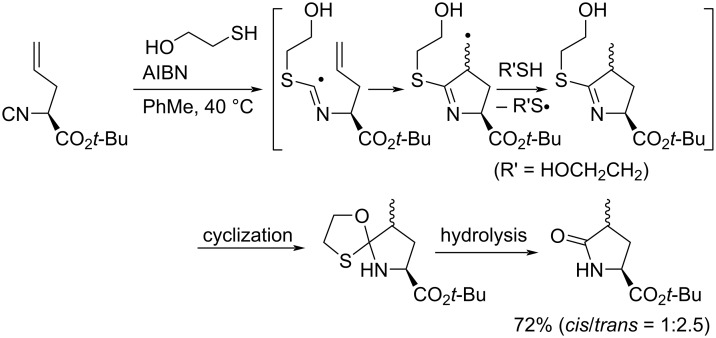
2-Thioethanol-mediated radical cyclization of alkenyl isocyanide.

At the same time, Bachi et al. succeeded in synthesizing a 5-membered nitrogen-containing heterocycle based on the 5-*exo* cyclization of isocyanides with alkenyl or alkynyl groups using thiols as mediators (Scheme 14) [57].

The generated thiyl radical attacks the isocyano group and forms imidoyl radical, which induces *5-exo* cyclization. The following hydrogen abstraction, intramolecular ionic cyclization, and hydrolysis during chromatography on silica gel affords the cyclic amide in good yield. They further applied this radical cyclization reaction as a key step in the synthesis of (±)-α-kainic acid [58].

**Scheme 15:**
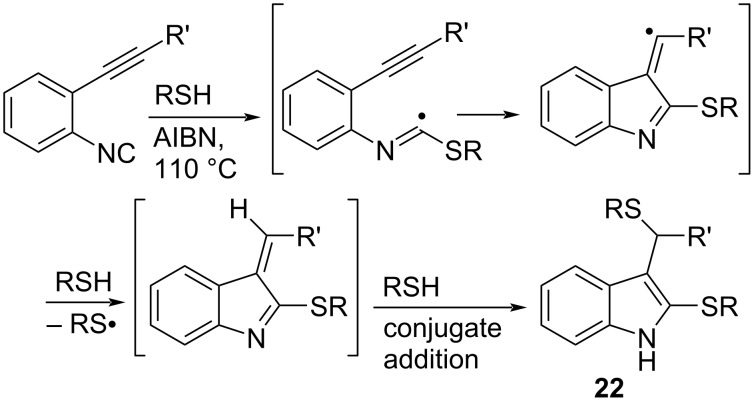
Thiol-mediated radical cyclization of *o*-alkenylaryl isocyanide.

Rainier et al. reported the thiol-mediated 5-*exo* cyclization of *o*-alkynylaryl isocyanides, which successfully afforded dithiolated indoles **22** (Scheme 15) [59]. However, depending on the reaction conditions, quinoline derivatives were also produced as byproducts (vide infra).

**Scheme 16:**
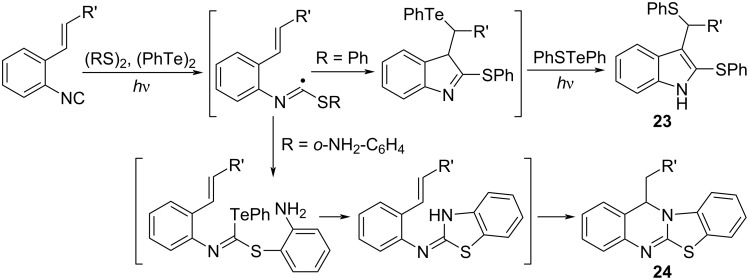
(PhTe)_2_-assisted dithiolative cyclization of *o*-alkenylaryl isocyanide.

The photoinduced reaction of *o*-ethenylaryl isocyanides with disulfides in the presence of diphenyl ditelluride yields the corresponding dithiolated indole derivatives **23** (Scheme 16) [60]. Initially, the thiotelluration products via 5-*exo* cyclization are formed in situ. The subsequent aromatization followed by photoinduced displacement of the PhTe group with the PhS group afford **23**. Furthermore, the photoinduced reaction of *ortho*-ethenylaryl isocyanides with bis(2-aminophenyl) disulfides affords tetracyclic compounds **24** in a single step.

**Scheme 17:**
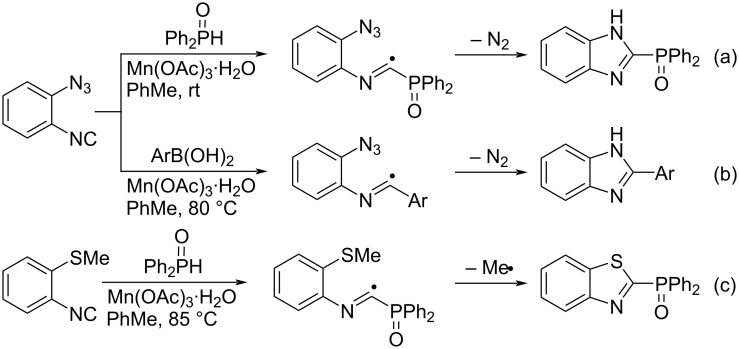
Trapping imidoyl radicals with heteroatom moieties.

Not only alkynyl and alkenyl groups, but also heteroatom moieties such as azido and sulfide groups can intramolecularly capture imidoyl radicals generated in situ, yielding the corresponding benzoimidazoles and benzothiazoles, respectively (Scheme 17). Upon treatment with Mn(OAc)_3_·H_2_O, diphenylphosphine oxide (Ph_2_P(O)H) and organoboron reagents (ArB(OH)_2_) generate Ph_2_P(O)• and Ar•, respectively, which add to isocyanides to form the imidoyl radicals. The capture of the imidoyl radicals with the azido group proceeds with the release of N_2_, and the amino radical formed abstracts hydrogen from the surroundings (Scheme 17a and 17b) [61]. The imidoyl radical formed by the addition of Ph_2_P(O)• to isocyanide can also be trapped intramolecularly by the methylthio group (Scheme 17c) [62].

**Scheme 18:**
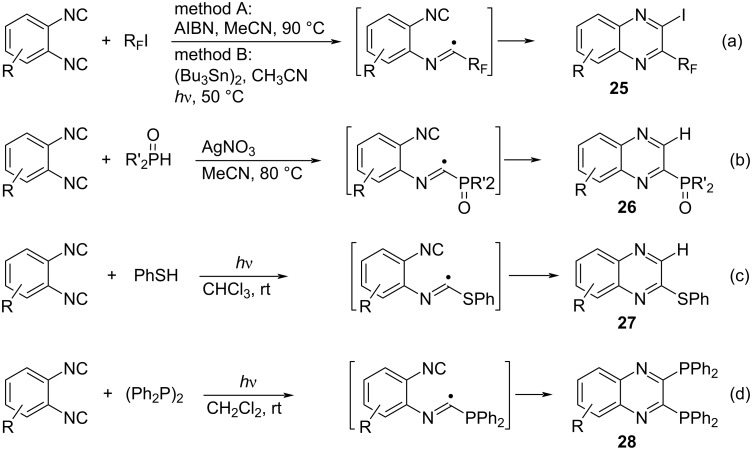
Trapping imidoyl radicals with isocyano group.

In the case of 1,2-diisocyanoarenes, the quinoxaline synthesis can be achieved via radical cyclization of imidoyl radical species, which proceeds by intramolecular capture with the *o*-isocyano group (Scheme 18) [63–66].

The iodoperfluoroalkylation with radical cyclization of *ortho*-diisocyanoarenes proceeded efficiently by using AIBN as initiator or using a hexabutyldistannane under visible light irradiation to afford the quinoxaline derivative **25** in good yields (Scheme 18a) [63]. At the same time, a similar quinoxaline synthesis was reported to proceed by irradiation with visible light in the presence of dibenzylamine ((PhCH_2_)_2_NH, MeCN, rt, blue LED) [64]. This reaction involves a visible-light-induced single electron transfer (SET) process. An efficient radical cascade cyclization has also been reported, in which a wide-range of 2-phosphoryl-substituted quinoxalines **26** were prepared in one pot via reaction of *ortho*-diisocyanoarenes with diarylphosphine oxides in the presence of AgNO_3_ (Scheme 18b) [65]. Chalcogen compounds such as PhSH and (PhSe)_2_ can be used as chalcogeno radical sources for the photoinduced radical cyclization of *ortho*-diisocyanoarenes to afford the corresponding 2-thiolated and 2,3-diselenated quinoxaline derivatives (e.g., **27**), respectively (Scheme 18c) [66]. Although diphosphines such as (Ph_2_P)_2_ and Ph_2_P(S)PPh_2_ did not intermolecularly add to isocyanides under radical reaction conditions (as mentiond above), they worked well for the photoinduced radical cyclization of *o*-diisocyanoarenes (Scheme 18d) [39]. The obtained quinoxaline-2,3-diphosphines **28** are promising ligands for transition metal catalysts such as palladium catalysts.

#### Aza-Bergman cyclization of *o*-alkynylaryl isocyanates

**Scheme 19:**
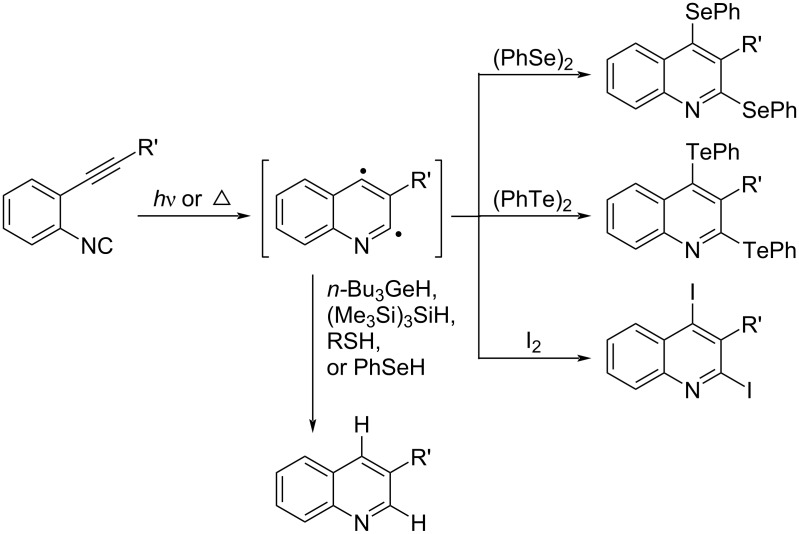
Quinoline synthesis via aza-Bergman cyclization.

In the case of *o*-alkenyl isocyanides, it has already been described that the 5-*exo* cyclization reaction proceeds selectively, yielding nitrogen-containing 5-membered cyclic products. In sharp contrast, when the photoirradiated reaction of *ortho*-alkynyl isocyanides was carried out, we found that an electrocyclization reaction, the aza-Bergmann cyclization, takes place, selectively yielding nitrogen-containing 6-membered cyclization products (Scheme 19) [67]. The aza-Bergmann cyclization of *ortho*-alkynyl isocyanides proceeds under milder conditions than the Bergmann cyclization of endiynes, forming quinoline-2,4-biradical intermediates. When diselenide, ditelluride, and diiodide are used as radical mediators, seleno, telluro, and iodo groups are introduced at the 2,4-positions of the quinoline, respectively [68–70].

On the other hand, in the case of germyl hydride, hydrosilane (TTMSS), selenol, and aliphatic thiols, hydrogen abstraction reaction by quinoline-2,4-biradical intermediates occurred to give 2,4-hydrogenated quinolines [69]. In the case of aromatic thiols, the ionic cycloaddition reaction proceeds to give 2-thiolated quinoline derivatives. In addition, the aza-Bergman cyclization can be thermally induced [71].

#### Radical cyclization of 2-isocyanobiarenes

**Scheme 20:**
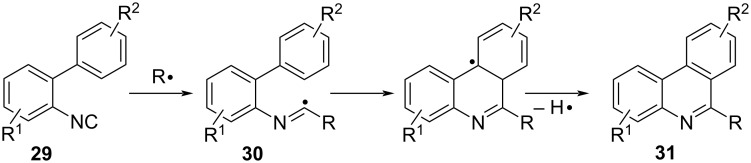
Phenanthridine synthesis via radical cyclization of 2-isocyanobiaryls.

The cycloaddition reaction with 2-isocyanobiaryls **29** under radical conditions is an excellent synthetic method for nitrogen-containing fused heterocycles such as phenanthridine derivatives **31** (Scheme 20) [72–73]. The reaction proceeds by addition of radical species to the isocyano group of **29** to form the imidoyl radical **30** as a key intermediate, which adds intramolecularly to the *ortho*-aryl group. The subsequent aromatization with the release of hydrogen (or proton) affords **31** in good yields.

**Scheme 21:**
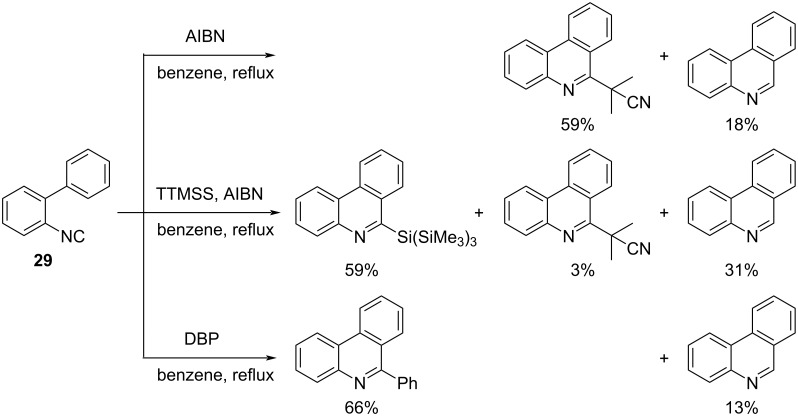
Phenanthridine synthesis by radical reactions with AIBN, DBP and TTMSS.

Nanni et al. reported the reaction of 2-isocyanobiphenyl with AIBN or dibenzoyl peroxide (DBP) affords 6-cyanoisopropyl- or 6-phenyl-substituted phenanthridine derivatives. When the reaction was performed in the presence of TTMSS and AIBN, 6-tris(trimethylsilyl)-substituted phenanthridine derivatives were mainly obtained (Scheme 21) [74].

**Scheme 22:**
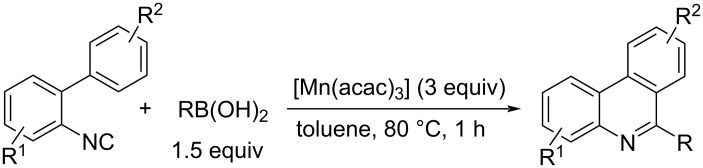
Phenanthridine synthesis by oxidative cyclization of 2-isocyanobiaryls.

Tobisu and Chatani et al. reported that a carbon radical generated by the reaction of RB(OH)_2_ with Mn(acac)_3_, added to isocyano groups, is leading to intramolecular cyclization with an *ortho*-aryl group. The formed aryl radical is oxidized by Mn(acac)_3_ to convert into an aryl cation, which can be deprotonated to synthesize tricyclic pyridine derivatives in a single step (Scheme 22) [75].

**Scheme 23:**
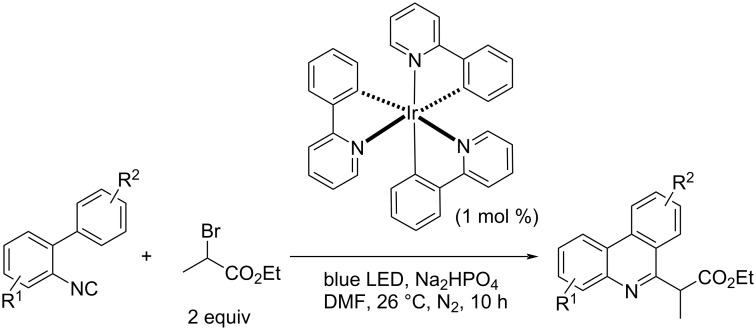
Phenanthridine synthesis using a photoredox system.

Zhang and Yu et al. also developed a cyclization of 2-isocyanobiaryls using a photoredox system [76–80] in which carbon radicals were generated by a photoredox reaction of α-bromopropanoates under visible light irradiation (Scheme 23) [81].

After these pioneering reports mentioned above, many examples of cyclization of 2-isocyanobiaryls using a metal-assisted system [82–86], photoredox system [21,87–90], or some other oxidation systems [91–93] were developed as excellent synthetic methods for nitrogen-containing fused heterocycles.

**Scheme 24:**
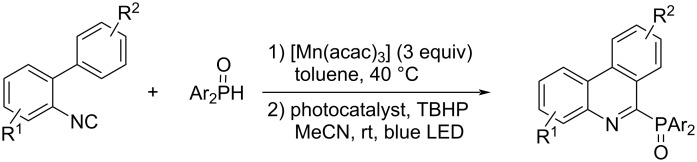
Phenanthridine synthesis induced by phosphorus-centered radicals.

On the other hand, there are not as many examples of reactions in which the addition of a heteroatom radical to 2-isocyanobiaryls generates an imidoyl radical intermediate to yield nitrogen-containing fused ring compounds. A few examples have been reported in which phosphorus-centered radicals generated from diarylphosphine oxides by Mn(OAc)_3_-assisted oxidation [94] or the photoredox system [95–97] were used in the radical cyclization reaction of 2-isocyanobiaryls (Scheme 24).

**Scheme 25:**
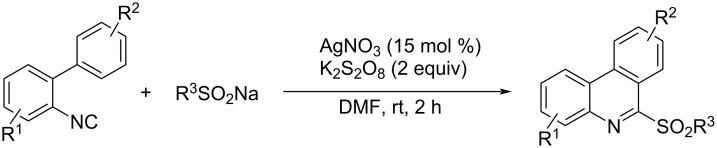
Phenanthridine synthesis induced by sulfur-centered radicals.

Yadav and Sigh et al. reported the direct synthesis of 6-sulfonylated phenanthridines via silver-catalyzed radical sulfonylation–cyclization of 2-isocyanobiphenyls (Scheme 25) [98].

**Scheme 26:**
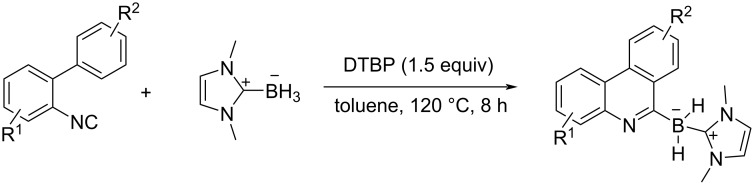
Phenanthridine synthesis induced by boron-centered radicals.

Wang et al. reported a radical borylative cyclization of 2-isocyanobiaryls with *N*-heterocyclic carbene borane (Scheme 26) [99]. The boryl radical generated via hydrogen abstraction in the presence of di-(*tert*-butylperoxy)-2-methylpropane (DTBP) as the radical initiator attacks isocyanide units, and the subsequent radical cyclization successfully proceeds to form a variety of borylated phenanthridines in moderate to good yields.

**Scheme 27:**
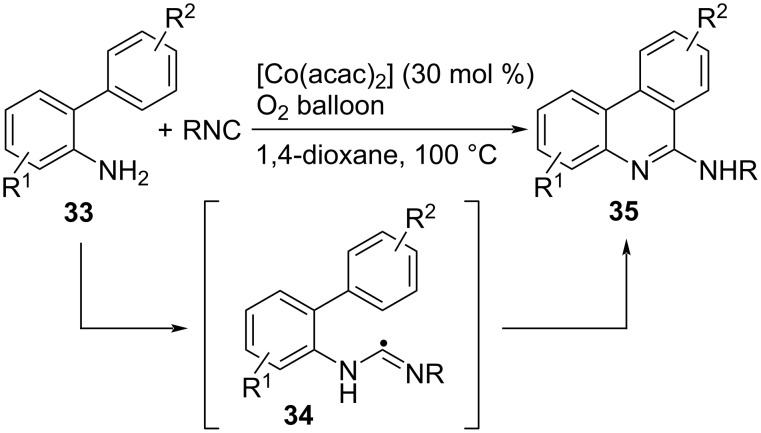
Phenanthridine synthesis by oxidative cyclization of 2-aminobiaryls.

Although the previous examples described the synthesis of phenanthridines via the radical addition of hetroatom radicals to 2-isocyanobiphenyl, another pathway to form phenanthridine scaffolds is the radical reaction of 2-aminobiaryls with free isocyanides. For example, oxidative generation of amino radicals from 2-aminobiaryls followed by intermolecular addition to isocyanides forms the imidoyl radicals, which undergo intramolecular cyclization and oxidative dehydrogenation to give phenanthridines (Scheme 27) [100].

## Conclusion

In this Perspective, the addition and cycloaddition reactions of heteroatom radicals with isocyanides have been described in detail and their synthetic application has been discussed. A number of useful synthetic reactions have been developed from the reactions of group 15 and 16 heteroatom radicals with isocyanides. On the other hand, the use of other heteroatoms in radical reactions with isocyanides has been limited due to the limitations of methods for generating these heteroatom radicals. It is highly expected that the radical reaction with isocyanides will be extended to many heteroatom radicals in the future, leading to the development of nitrogen-containing functional molecules modified with a variety of heteroatom functional groups. We hope that this Perspective will help in the development of such new reactions.

## Data Availability

Data sharing is not applicable as no new data was generated or analyzed in this study.
